# Surface Properties of Eggshell Powder and Its Influence on Cement Hydration

**DOI:** 10.3390/ma15217633

**Published:** 2022-10-30

**Authors:** Yinghou He, Dehao Che, Xiaowei Ouyang, Yanfei Niu

**Affiliations:** 1 Research Center of Wind Engineering and Engineering Vibration, Guangzhou University, Guangzhou 510006, China; 2 School of Civil Engineering, Guangzhou University, Guangzhou 510006, China

**Keywords:** eggshell powder, surface chemical properties, zeta potential, hydration, cement paste, C–S–H

## Abstract

Using eggshell powder (EP) to replace partial cement in cement-based materials can abate pollution caused by eggshell discard and cement production. In this paper, the surface property of EP and its influence on cement hydration were studied. Quartz powder (QP) and limestone powder (LP) were used as references. First, the chemical composition of EP was characterized. Then, the surface charge properties of these materials were analyzed using zeta potential measurement. The interactions between EP surface and Ca^2+^ were discussed based on the zeta potential test. Afterward, a scanning electron microscope (SEM) was applied to observe the morphology of hydrates on the surfaces of these materials. The results indicated that, although the compositions of EP and LP are similar, the surface charge properties are significantly different. This is likely due to the existence of organic matter on the surface of EP and the difference in the atomic structure. As shown from the zeta potential test, EP exhibits similar interaction with Ca^2+^ as QP. The interactions between EP surface and Ca^2+^ are much weaker than that between LP and Ca^2+^. These weak interactions lead to the growth of C–S–H on the surface of EP particles less than that of LP particles. The chemical reactivity of EP can be improved by using heat treatment, electrical oven, etc. This study will provide theoretical support for the better use of EP in cement-based materials.

## 1. Introduction

Cement production is one of the main causes of environmental problems due to the high consumption of energy and non-renewable mineral resources, and high carbon dioxide emission [1,2,3,4,5,6,7,8]. In order to promote the sustainable development of the cement industry, effective measures to reduce the consumption of energy and non-renewable mineral resources have become an important topic in the field of cement research. Therefore, the feasibility of replacing part of the cement with various fillers, such as calcium-based stone powder, has been extensively studied [9,10,11,12,13,14]. In considering these calcium-based stone powders, eggshell powder (EP) is often overlooked. Millions of eggshells were discarded every day. Eggshell has been listed as one of the environmental problems [15,16,17]. Furthermore, if the waste eggshell is simply buried in landfills, the corrosion of the film on the eggshell will attract pests and lead to the spread of disease [18,19]. Nandhini and Karthikeyan [20] state that the disposal of solid waste is a challenging issue faced by developing countries. For instance, India generates about 3.8 billion kilograms of eggs annually. Moreover, the increase in domestic consumption would further contribute to larger solid waste generation. Lately, Chen et al. [21] conducted a study to demonstrate the feasibility of using bio-waste eggshell powder as a filler in cement. Different EP amounts were tested to investigate its filler effect. The test results found that cement hydration was visibly increased, which was also supported by the findings from decoupled cumulative hydration heat curves and the microscopic observations. The sustainable assessment revealed that a 5-percent EP replacement exhibited the best performance and reduced environmental pollution. As pointed out by Yang et al. [22], Eggshell powder has the potential to act as a partial substitute for cement and fine aggregate. Thus, by using EP, it is expected to produce a more environmentally friendly construction material. Substituting part of the cement with EP is of great significance to the energy-saving and emission reduction of the cement industry and the large-scale resource utilization of eggshells.

Eggshells have such great potential in the application of cement-based materials that their use has attracted the attention of many researchers [23,24,25,26,27,28,29]. Jhatial et al. [30] reported that the eggshell can be used as auxiliary cementitious material after being ground into powder. The best cement replacement amount is 10% to achieve the maximum compressive strength, regardless of the fineness of the eggshell powder. Jaber et al. [31] measured the water absorption, thermal conductivity, compressive strength and hardness properties of mortar specimens after curing for 28 d. It was found that adding EP can improve the physical and mechanical properties of cement mortar. The mortar heated at 750 ℃ for 1 h in an electric furnace has better performance. Ofuyatan et al. [32] reported that partial substitution of cement with EP improves fluidity and workability. In addition, microstructure analysis showed that partial substitution of cement with EP improves the interface interaction between different components of concrete. Pliya and Cree [33] tested the compressive strength and flexural strength of mortar specimens containing limestone powder and eggshell powder. The results showed that limestone has a better performance than eggshell powder. When 5 wt.% eggshell powder is added, its strength is slightly lower than that of limestone and control mortar. Most recently, Yang et al. [22] discussed the mechanical properties of cementitious materials containing EP by using a scientometric analysis method, and the effects of EP on the performance of the cementitious materials were investigated. The results suggested that 25% EP in cementitious materials is beneficial for material performance. Dewangan et al. [34] proposed a novel approach to using injectable macroporous apatite bone cement under physiological conditions. Its solid phase consists of hydroxyapatite and β–tricalcium phosphate (derived from eggshell) and the liquid phase contains the biopolymeric solution and disodium hydrogen phosphate. The developed eggshell-derived apatite bone cement could act as a potential material for repairing bearing defects in orthopedic applications. Amin et al. [35] considered both sugarcane bagasse ash (SCBA) and nano eggshell powder (NEP) as cementitious materials and added them to the cement. It was found that the setting time of high-strength concrete was accelerated by adding NEP while delayed by increasing SCBA. The experimental results showed that the optimum proportion of the mixture was 5% NEP with 15% SCBA. Kumar et al. [36] conducted the replacement of cement with fly ash of 20%, while EP from 0 to 15% and properties of the cementitious material were evaluated at the ages of 1, 7 and 28 days. The findings indicated that a cement mixture with 10% eggshell and 20% fly ash showed better performance. Grzeszczyk et al. [37] performed experimental studies of adding EP instead of limestone in cement. Different ratios of EP (by weight) were added to Portland cement (CEM I 42.5 R) and the phase composition of the eggshell was determined using an XRD technique and IR/Raman spectroscopy. Because of the impact of eggshell admixtures, the hydration of cement paste was delayed and the strength parameters of mortar were reduced slightly.

Up to now, much research has been done on the eggshell powder used in cement-based materials. The eggshell was widely perceived as a kind of limestone filler. It is worth mentioning that most of the studies are focused on the macroscopic properties of cement-based materials mixed with eggshell powder. However, the performance improvement of cement filled with partial eggshell powders is not thoroughly understood and validated from the micro level of the cement mixture. Thus, the surface property of eggshell and its effect on cement hydration have rarely been addressed. Therefore, this research will explore the surface property of the EP and its influence on cement hydration at the micro-nano scale.

In this study, quartz powder and limestone powder were used as reference materials. First, XRD, TGA and FTIR were used to determine the composition of the eggshell powder. Then, the zeta potential test was used to study the particle surface properties of the three powders. After that, the morphology of hydrates on the surfaces of eggshell powder, quartz powder and limestone powder was observed with a scanning electron microscope. The chemical properties of hydration products were studied. Finally, the mechanism of the effect of surface characteristics of EP on cement hydration was investigated.

## 2. Materials and Methods

### 2.1. Materials

**Figure 1:**
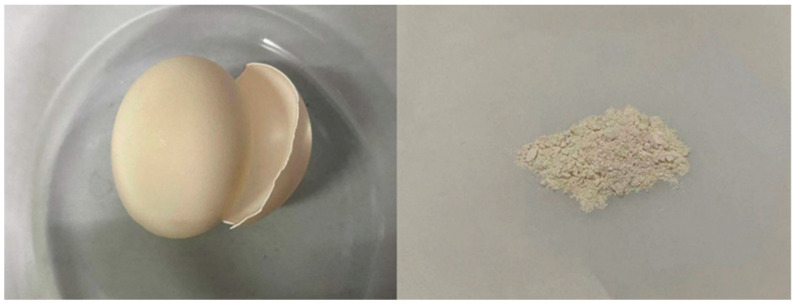
Raw eggshells and eggshell powder.

The cement used in this experiment is Portland cement type I (PC). Eggshell powder (EP) is obtained by removing the eggshell membrane from the collected eggshell, then drying it at a temperature of 45 °C in an oven for about 2 h and grinding it into powder, as shown in Figure 1.

**Figure 2:**
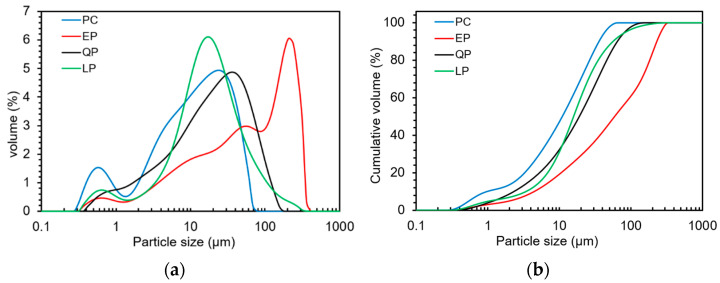
Volume distribution (**a**) and cumulative distribution (**b**) of particle size of EP, QP, LP and cement.

Quartz powder (QP) was purchased from Heyuan Zhaochuan Quartz Calcium Industry Co., Ltd., China. Limestone powder (LP) was purchased from Jingmen Shunzhan Calcium Industry Co., Ltd., Jingmen, China. The particle sizes of PC, EP, QP and LP powders were measured by a laser diffraction particle size analyzer (Malvern, Mastersizer 2000, Malvern, UK), as given in Figure 2. It can be seen that PC, QP and LP have similar particle size while EP is slightly larger.

The chemical compositions of EP, QP and LP are listed in Table 1. Note that the main composition of QP is SiO_2_ while the chemical compositions of EP and LP are not; both EP and LP contained more than 96% CaCO_3_.

Although the main compositions of EP and LP are similar, many studies have shown that the organic matrix content of eggshells is about 2–3% [38,39,40,41,42]. As shown in Table 2, the organic element content of EP was measured with an organic element analyzer (Vario MACRO cube). It indicates that EP contains organic matter.

**Figure 3:**
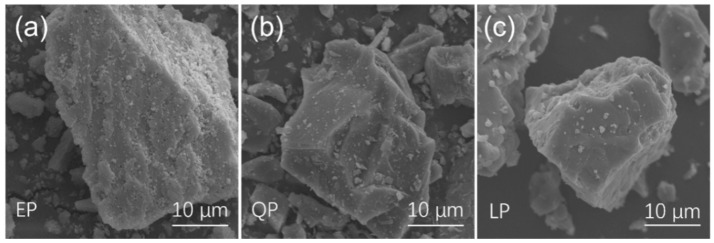
Surface morphologies of EP (**a**), QP (**b**) and LP (**c**).

Figure 3 gives the original morphology of EP, QP and LP particles. It can be seen that there is no obvious difference in the original shape of the three kinds of particles. All three are irregular polyhedrons. It is noteworthy that the difference between EP particles and QP and LP particles is that the EP particles have many small holes at their surface.

**Figure 4:**
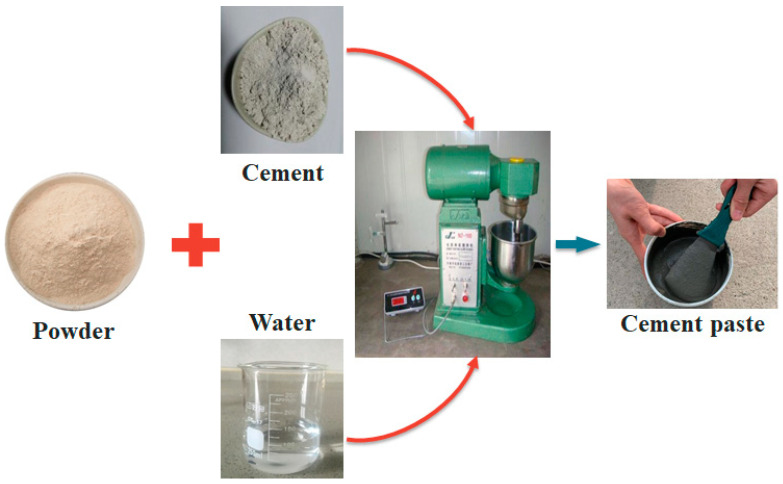
Production of cement paste.

The typical production process of cement paste with various powders in the laboratory is shown in Figure 4. Correspondingly, the mix proportion of cement pastes are listed in Table 3. These samples were prepared in a Hobart mixer following the standard procedures described in ASTM C305 [43]. The composite cementitious materials mixed with EP, QP and LP are named EP40, QP40 and LP40, respectively.

### 2.2. X-ray Diffraction Analysis (XRD)

With the aim of studying the phase composition of EP, EP40, QP40 and LP40, XRD analysis was applied with an X-ray powder diffractometer (PANalytical, PW3040/60, Malvern, UK). About 5 g powder was taken to make a sample and then measured by CuKα radiation (λ = 1.5418 Å). The scanning angle of the sample ranges from 2θ = 5° to 2θ = 80° and the step size is 0.02°.

### 2.3. Thermogravimetric Analysis (TGA)

The TGA data of EP, EP40, QP40 and LP40 were measured from 50 °C to 800 °C using a thermogravimetric analyzer (PerkinElmer, TGA4000, Waltham, MA, USA). The heating rate is 10 °C/min, and the protective gas used in the test is N_2_.

### 2.4. Fourier-Transform Infrared (FTIR) Spectroscopy Analysis

A FTIR spectrometer (Bruker, TENSOR II+ Hyperion2000, Billerica, MA, USA) was used to measure the infrared spectrum data of EP, EP40, QP40 and LP40. The spectral range is 450–4000 cm^−1^. The spectral resolution is 0.4 cm^−1^; the wavenumber accuracy is 0.01 cm^−1^; and the signal-to-noise ratio is 45,000:1.

### 2.5. Zeta Potential Test

The surface charge of particles in solution is affected by the number of ions adsorbed on the surface of particles. Zeta potential can be used to measure the surface charge of particles suspended in solution. Once contacted with water, the cement particles will dissolve and release various ions. Particles in the solution adsorb the ions and the particle surface presents a positive or negative charge. The surface chemical properties of EP, QP and LP can be characterized with zeta potential measurement using a Zetasizer Nano ZS (Malvern Instruments Ltd., Malvern, UK). To do the test, five sets of simulated solutions are configured. The first group is a Ca(OH)_2_ solution with a concentration ranging from 0.2 mmol/L to 20 mmol/L. The second and third groups are solutions composed of Ca(OH)_2_ and NaOH or KOH. The concentration of Ca(OH)_2_ is 0.1–8 mmol/L, while that of NaOH and KOH are 50 mmol/L, respectively. The fourth group is a mixed solution of Ca(OH)_2_ and K_2_SO_4_. The concentration of Ca(OH)_2_ is 0.1–19.6 mmol/L, and K_2_SO_4_ is classified into two concentrations: 10 and 50 mmol/L, respectively.

### 2.6. SEM Analysis

The morphology of hydrates on the particle surface was observed with a Phenom–ProX electron microscope (FEI, Hillsboro, OR, USA). The mixtures of the sample are shown in Table 3. The sample preparation process of the hydrated product morphology is as follows: In the hydration time (15 min, 4 h, 7 h), a certain amount of cement paste (1 g) was taken and put into absolute ethanol to stop the hydration. After the termination of hydration, the sample was filtered and dried in a vacuum drying oven. Then, the samples were stored in a vacuum box until used. SEM observation was performed on the samples coated with gold. The acceleration voltage was 15 kV, and the SED model was used.

## 3. Results and Discussion

### 3.1. Chemical Compositions of EP

#### 3.1.1. XRD Analysis

**Figure 5:**
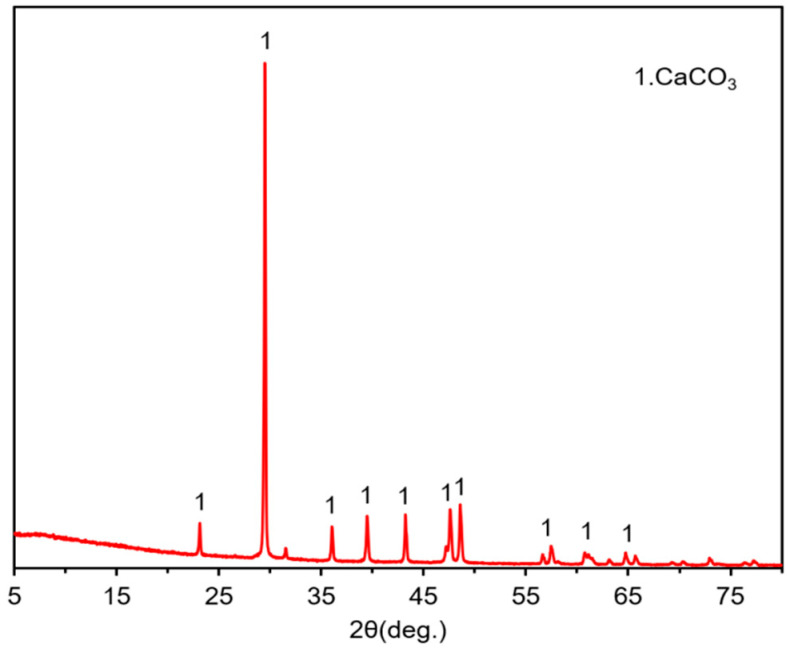
X-ray diffraction (XRD) analysis of EP.

Figure 5 shows the XRD pattern of EP. It can be seen that the phases corresponding to the diffraction peaks of EP are all CaCO_3_, which is similar to the XRD pattern of limestone [44,45]. This indicates that the main component of EP is the same as that of LP.

#### 3.1.2. TGA Analysis

**Figure 6:**
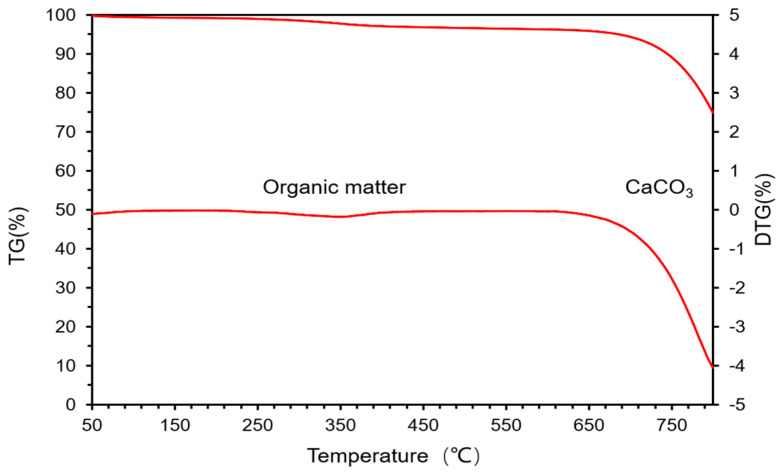
Thermal decomposition of EP by TGA–DTG analyses.

As shown in Figure 6, the thermal decomposition diagram of EP was obtained by TGA–DTG analysis. It can be seen that a slight weight loss exists between 250 °C and 400 °C, corresponding to the decomposition of organic matter in EP. Calculated from the TGA–DTG results, the organic content in EP is about 3%. The continuous severe weight loss after 600 °C is the weight loss caused by the decomposition of a large amount of CaCO_3_ contained in EP.

#### 3.1.3. FTIR Analysis

**Figure 7:**
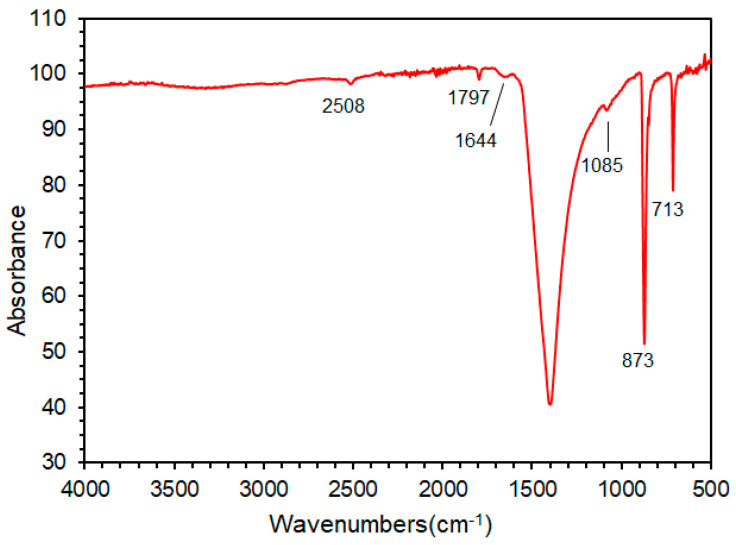
FTIR spectroscopy analysis of EP.

Figure 7 gives the FTIR spectrum of EP. The three bands of carbonate-based asymmetric stretching (ν3), out-of-plane bending (ν2) and in-plane bending (ν4) correspond to 1397 cm^−1^, 873 cm^−1^ and 713 cm^−1^, respectively. The two low-intensity bands are at 2508 and 1797 cm^−1^ [46]. The wide band at 1644 cm^−1^ refers to the amide-related carbonyl group (-C=O stretching), and the band at 1085 cm^−1^ corresponds to the asymmetric stretching of the phosphate group [47]. This is similar to the results obtained by XRD and TGA. The results of XRD, TGA and FTIR show that the main component of EP is CaCO_3_ and contains a small amount of organic matter.

### 3.2. Zeta Potential Test

#### 3.2.1. Effect of Ca^2+^ Concentration

**Figure 8:**
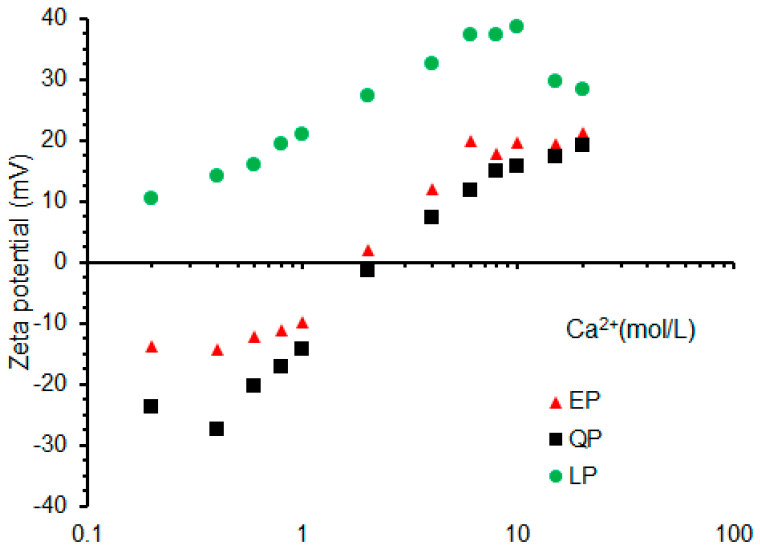
Zeta potential of EP, QP and LP in Ca (OH)_2_ solutions with different Ca^2+^ concentrations.

The zeta potential of EP, QP and LP particles in Ca(OH)_2_ solution with concentrations from 0.2 to 20 mmol/L is shown in Figure 8. It is noted that the initial potential of LP is positive. With the increase of Ca(OH)_2_ concentration, the potential value increases gradually. Compared with LP, QP has a lower potential under the same concentration of Ca(OH)_2_ solution. The silanol group in QP powder began to dissolve after contacting with the solution [48,49]:

≡SiOH + H^+^ ⇌ ≡SiOH_2_^+^ (1)

≡SiOH + OH^−^ ⇌ ≡SiO^−^ + H_2_O (2)

The initial potential of QP particles is negative due to the formation of SiO^−^ ions in the solution. With the increase of Ca^2+^ concentration in solution, more Ca^2+^ is adsorbed on the surface of QP particles, which could compensate for the negative potential produced by ionization. When Ca^2+^ concentration reached about 2 mmol/L, the QP reached the zero potential point.

According to Table 1, EP and LP have similar chemical compositions. However, EP exhibits a potential change characteristic similar to QP. The initial potential is negative and reaches the zero potential point when the Ca^2+^ concentration is approximately 2 mmol/L. This may be attributed to the organic matter on the EP particle surfaces, which is adverse to the adsorption Ca^2+^.

#### 3.2.2. Effect of Na^+^ and K^+^ Concentration

**Figure 9:**
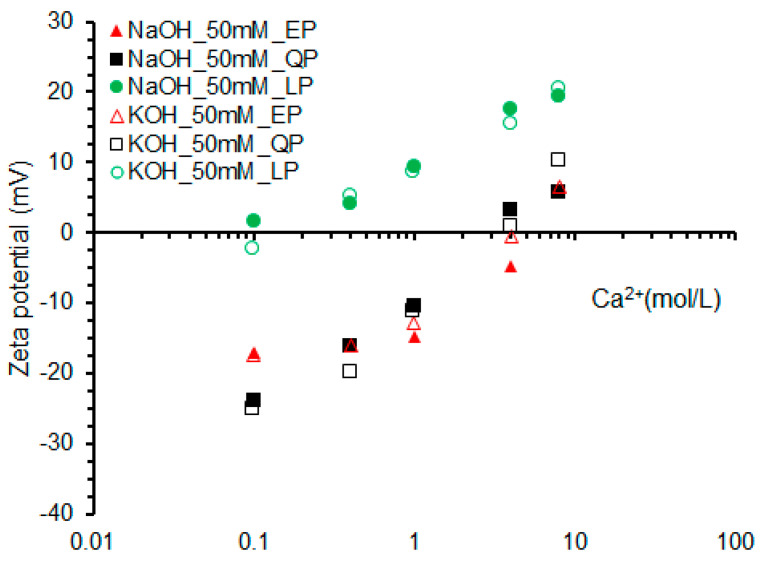
Zeta potentials of EP, QP and LP with Ca^2+^ concentration in 50 mmol/L NaOH and KOH solutions.

The zeta potential evolution of EP, QP and LP in NaOH and KOH solutions with the change of Ca^2+^ concentration is shown in Figure 9. As can be observed, with the same concentration of Ca^2+^, the potential value of LP is higher than that of EP. The zero potential point of EP reached a 4 mmol/L (Ca^2+^ concentration), which is larger than the zero potential point in a Ca(OH)_2_ solution. Additionally, the zero potential of QP particles is also higher than 2 mmol/L. This is because in a higher alkaline solution, the more SiO^−^ is generated, the more Ca^2+^ is needed to compensate for the negative potential. EP shows a potential change trend similar to QP in NaOH and KOH solutions.

#### 3.2.3. Effect of SO_4_^2−^ Concentration

**Figure 10:**
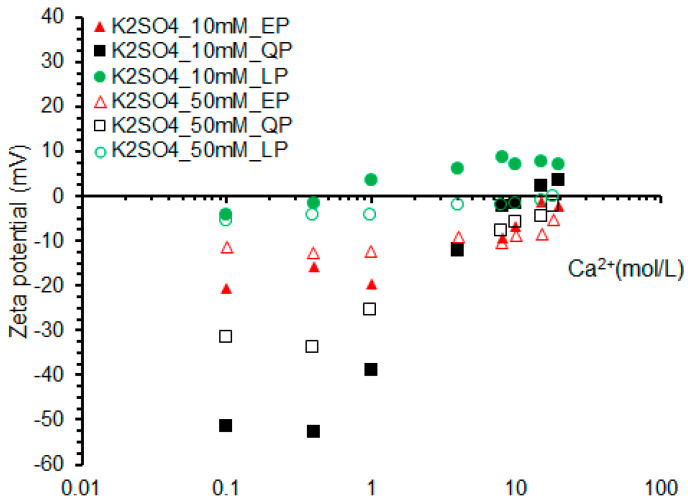
Zeta potentials of EP, QP and LP with Ca^2+^ concentration in 10 mmol/L and 50 mmol/L K_2_SO_4_ solutions.

Figure 10 shows the zeta potential changes of EP, QP and LP in the mixed solution. In 10 mmol/L K_2_SO_4_ solution, the zero potential point of QP is that the concentration of Ca^2+^ reaches 10 mmol/L. However, EP has not reached the zero potential point in solution. This indicates that the adsorption of EP to SO_4_^2−^ is slightly stronger than that of QP at high Ca^2+^ concentration. In 50 mmol/L K_2_SO_4_ solution, the zeta potentials of EP and QP are always negative, which is due to the higher concentration of SO_4_^2-^ in the solution than that of Ca^2+^. As mentioned above, due to the strong adsorption capacity of LP for Ca^2+^, in 10 mmol/L K_2_SO_4_ solution, the zero potential point of LP is about 1 mmol/L of Ca^2+^. While in the case of concentration of K_2_SO_4_ up to 50 mmol/L, the LP needs 15 mmol/L Ca^2+^ to reach the zero potential point.

### 3.3. Morphology of Hydration Products on the Surface of EP, QP and LP

**Figure 11:**
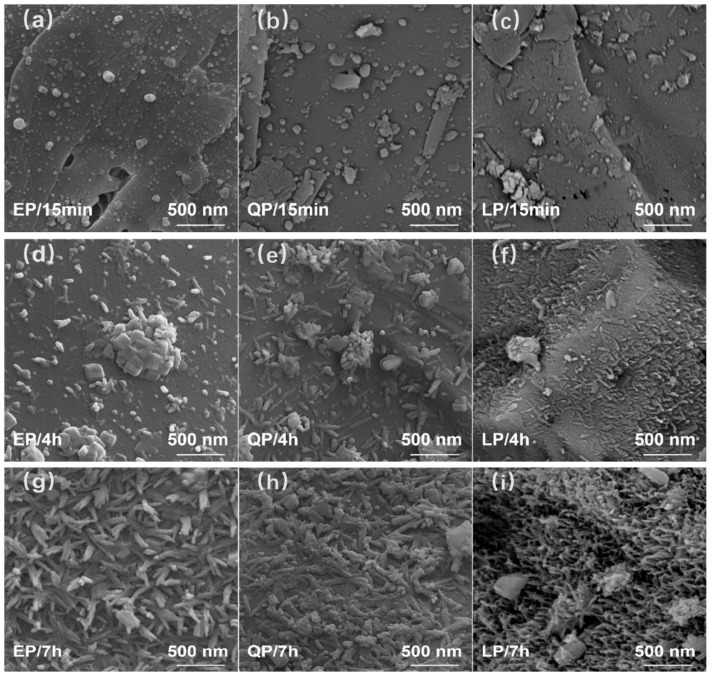
Morphology of hydration products on the surface of EP particle at 15 min (**a**), 4 h (**d**) and 7 h (**g**), QP particle at 15 min (**b**), 4 h (**e**) and 7 h (**h**), and LP particle at 15 min (**c**), 4 h (**f**) and 7 h (**i**).

The appearance of surface hydration products of EP, QP and LP particles corresponding to various hydration times (15 min, 4 h, 7 h) is shown in Figure 11. Figure 11a–c respectively show the surface of EP, QP and LP particles after hydration for 15 min. Note that there is no hydration product on the surfaces of EP and QP particles. However, a small amount of needles like C–S–H grew on the surface of LP particles. Figure 11d–f respectively give the surface morphology of EP, QP and LP particles after 4 h hydration. The C–S–H on the EP and QP particle surfaces grew into needle shape gradually. The hydration products on the surface of LP particles have grown into a layer covering the surface of particles after 7 h of hydration, as shown in Figure 11g–i. The needle-shaped C–S–H on the surface of EP and QP particles continues to grow. It is worth noting that C–S–H on the EP and QP particle surfaces is disordered and not dense. The surface of LP particles formed an orderly dense C–S–H layer after 7 h hydration. Moreover, the C–S–H on the surface of LP particles is perpendicular to the particle surfaces. From the results of the morphological characteristics of the surface hydration products on these three kinds of particles at different hydration times, EP and QP showed similar hydration product formation. Under the same hydration time, the distribution of hydration products on the surface of LP particles is more orderly and denser than that of EP and QP particles. This further shows that although the composition of EP is similar to that of LP, it shows similar characteristics to QP.

### 3.4. Chemical Properties of Hydration Products

#### 3.4.1. XRD Analysis

**Figure 12:**
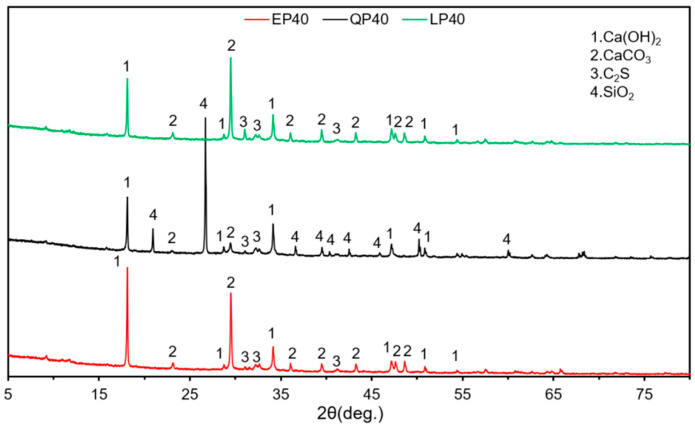
X-ray diffraction (XRD) analysis of EP40, QP40 and LP40 at 7 d.

**Figure 13:**
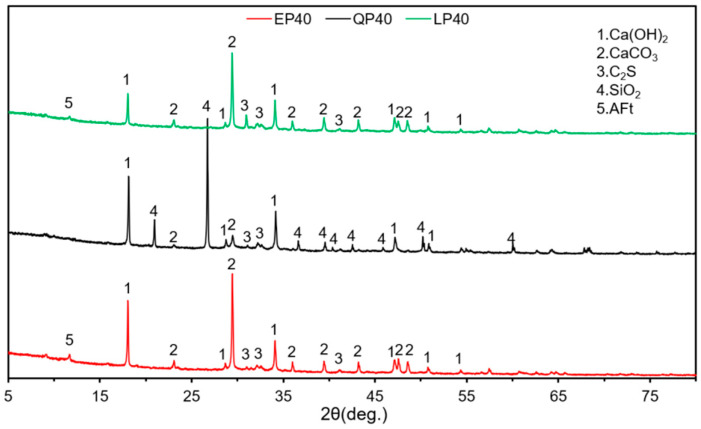
X-ray diffraction (XRD) analysis of EP40, QP40 and LP40 at 28 d.

Figure 12 and Figure 13 give the XRD patterns of EP40, QP40 and LP40 after hydration for 7 and 28 d, respectively. Note that the main components of EP40 and LP40 are Ca(OH)_2_, CaCO_3_ and C–S–H after 7 d of hydration. The main components of QP40 are SiO_2_, Ca (OH)_2_, CaCO_3_ and C–S–H. With the hydration time up to 28 d, the composition of cement paste remains unchanged, and the diffraction peak intensity of each substance does not change significantly. The main components of QP40 are similar.

#### 3.4.2. TGA Analysis

**Figure 14:**
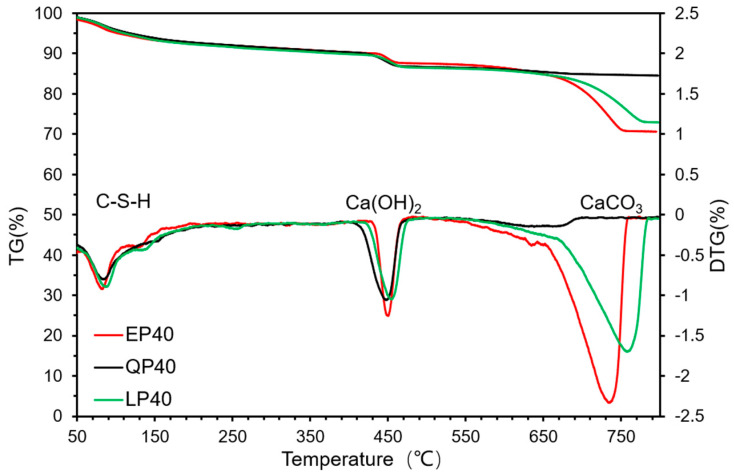
Thermal decomposition of EP40, QP40 and LP40 by TGA–DTG analyses at 7 d.

**Figure 15:**
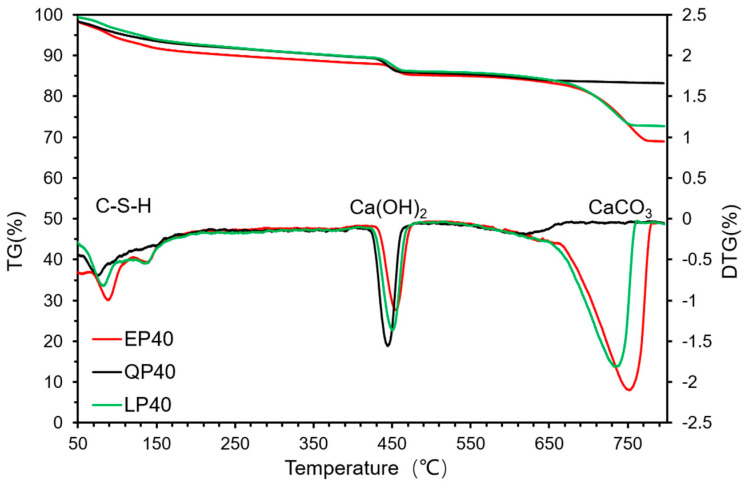
Thermal decomposition of EP40, QP40 and LP40 by TGA–DTG analyses at 28 d.

Figure 14 and Figure 15 show the TGA–DTG analysis results of EP40, QP40 and LP40 after 7 and 28 d of hydration. The first weight loss was recorded at 50 °C to 150 °C, which was attributed to the dehydration and evaporation of water in C–S–H. The second obvious weight loss occurred between 410 °C and 490 °C due to the decomposition of Ca(OH)_2_. In the range of 650–800 °C, the weight loss of EP40 and LP40 is obvious, which is caused by the CaCO_3_ contained in EP and LP.

#### 3.4.3. FTIR Analysis

**Figure 16:**
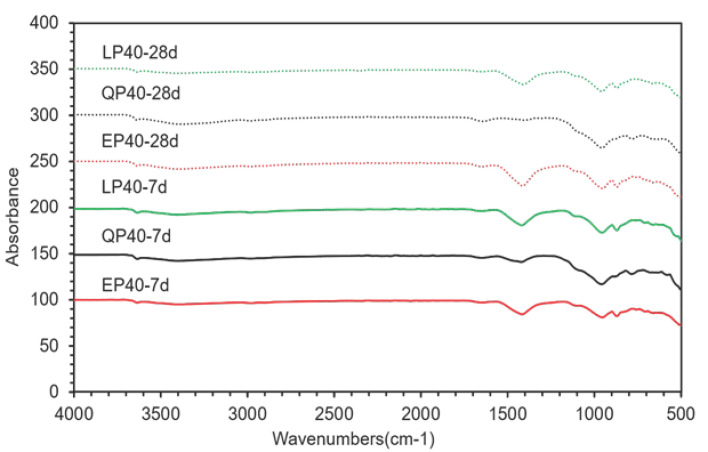
Fourier transform infrared spectroscopy analysis (FTIR) of EP40, QP40 and LP40.

Figure 16 demonstrates the FTIR spectra of EP40, QP40 and LP40 at 7 and 28 d. The band at 3640 cm^−1^ corresponds to the OH^−^ group in Ca(OH)_2_ [50]. Monocarboaluminate is related to the split ν3-CO_3_^2−^ at approximately 1420 cm^−1^ with ν2-CO_3_^2−^ at approximately 880 cm^−1^. The intensity found between 900 and 1100 cm^−1^ is associated with the formation of C–S–H [51]. The results obtained are consistent with those of XRD and TGA.

## 4. Discussion

To characterize the chemical composition of EP, XRD, TGA and FTIR were used. Through XRD analysis, it can be known that the main component of EP is CaCO_3_, which is the same as LP. Furthermore, the results of TGA and FTIR showed that EP contained a small amount of organic matter. The surface chemical properties of particles are closely related to the interaction between particles and ions, which has an important influence on the formation of hydration products on the particle surface [52,53,54,55]. The powder particles adsorb various free ions in the cement pore solution, which makes the particle surface present positive and negative charges. The surface chemical properties of the particles were examined by the zeta potential test. The results of zeta potential show that in the four simulated solutions configured, the potentials of EP and LP are very different, while EP and QP show similar potential results. This shows that even though EP and LP have similar chemical compositions, EP particles have similar surface charge properties as QP particles.

As illustrated in Figure 7, the zeta potential of EP is lower than that of LP and is similar to QP. The zero potential point of EP in Ca(OH)_2_ solution is about 2 mmol/L Ca^2+^, which infers that the adsorption capacity of EP particles for Ca^2+^ is weaker than that of LP particles. Studies [56] have shown that Ca^2+^ is closely related to the nucleation and growth of C–S–H. Since the zeta potential results show that EP has a weaker Ca^2+^ adsorption capacity than LP, the nucleation of C–S–H at the surface of EP particles is less than that at the surface of LP particles, as indicated in Figure 11. The growth of C–S–H on the EP particle surfaces is less than that of LP during all hydration times (15 min, 4 h, 7 h). After 7 h of hydration, the C–S–H on the surface of EP particles did not form the same dense structure as the surface of LP particles. This may be due to the organic matter on the EP particle surfaces and the difference in the atomic structure, which is unfavorable for Ca^2+^ absorption and thus the nucleation and growth of C–S–H.

It was reported that the addition of eggshell powder to Portland cement paste accelerates hydration due to its chemical reaction and nucleation sites [18]. In our study, it can be observed that the surface of EP can serve as the nucleation site, promoting cement hydration, but with less effectiveness than LP. The results of XRD and TGA of the cement paste incorporating fillers (i.e., EP, QP and LP) showed that the main hydration products of EP40, QP40 and LP40 are similar and the amount of the hydrates are not obviously different. It was suggested that the chemical reactions of EP in cement paste are not significant. It is possible due to the different treatments for EP production. In our study, EP was obtained by removing the eggshell membrane from the collected eggshell, then drying it at a temperature of 45 °C in an oven for about 2 h and grinding it into powder. In some reported studies [18,57,58], the eggshell was dried at a temperature of 120 °C, or higher temperature, or using an electrical oven. These treatments would affect the chemical activity and the surface properties of EP, thus its performance in cement hydration.

## 5. Conclusions

The chemical composition of EP was first analyzed in this study. Then, the surface charge properties of EP particles were investigated through comparing with QP and LP. The morphology of hydration products on the EP particle surfaces and the chemical composition of long-term hydration products were studied. Through the analysis of the experimental results, the main findings and conclusions can be summarized:

(1) The main components of EP and LP are more than 96% CaCO_3_. Although the compositions of EP and LP are similar, the surface charge properties are significantly different. This is likely due to the existence of organic matter on the surface of EP and the difference in the atomic structure. The adsorption capacity of EP for Ca^2+^ is similar to that of QP but weaker than that of LP.

(2) In different hydration times (15 min, 4 h, 7 h), the nucleation and growth of C–S–H on the EP particle surfaces are less than that of LP. The formation of hydration products on the surface of EP particles is similar to that of QP particles, which is in agreement with the adsorption capacity for Ca^2+^.

(3) The main hydration products of EP40, QP40 and LP40 are similar and the amount of the main hydrates are not obviously different. This indicates that the chemical reactions of EP in cement paste are not significant.

(4) This study was performed using an EP with low chemical activity. The eggshell treated with high temperature would affect the chemical activity and the surface properties of EP, thus its performance in cement hydration. This needs to be addressed in future study.

## Figures and Tables

**Table 1:** Chemical composition of EP, QP and LP (% by mass).

Type	CaCO_3_	SiO_2_	Al_2_O_3_	Fe_2_O_3_	CaO	MgO	K_2_O	Na_2_O
EP	97.37	0.37	0.06	0.41	-	0.85	0.10	0.27
QP	-	98.50	0.89	0.15	-	-	0.45	-
LP	96.09	0.15	-	0.15	-	-	0.01	0.02

**Table 2:** Organic element composition of EP (%).

Type	N	C	H	S	O
EP	0.1	12.31	2.52	0	-

**Table 3:** Mix proportion of cement pastes.

Mixture	Cement (%)	EP (%)	QP (%)	LP(%)	w/b
EP40	60	40	-	-	0.4
QP40	60	-	40	-	0.4
LP40	60	-	-	40	0.4

## Data Availability

Not applicable.
